# Sheep dietary preferences in targeted grazing: demographic, management, and weather effects in northern mixed-grass prairie

**DOI:** 10.3389/fvets.2024.1502948

**Published:** 2024-11-28

**Authors:** Aaron J. Kersh, Hannah M. Fraley, J. Derek Scasta, Justin D. Derner, Paulo de Mello Taveres Lima, Whit C. Stewart

**Affiliations:** ^1^Department of Animal Science, University of Wyoming, Laramie, WY, United States; ^2^Department of Ecosystem Science and Management, University of Wyoming, Laramie, WY, United States; ^3^USDA-Agricultural Research Service, Rangeland Resources and Systems Research Unit, Cheyenne, WY, United States

**Keywords:** Dorper, fecal DNA metabarcoding, Rambouillet, sheep dietary preference, targeted grazing

## Abstract

Diet selection and composition of sheep target grazing plains larkspur (*Delphinium geyeri* Greene) in northern mixed-grass prairie were evaluated during a drought year (2022). Thirteen Rambouillet ewes (3-to 6-year-old, body weight (BW) 76 kg ± 2.9), 14 Dorper ewes (3-to 6-year-old, BW 47 kg ± 1.8), and 123 Dorper ram lambs (<1 year-old, BW 25 kg ± 0.4) were used for targeted grazing. Over the 20-day first phase (mid-May to early June), sheep were subjected to three stock density treatments: (1) high, 40 animal units (AU)/ha, (2) moderate, 20 AU/ha, and (3) light, 13 AU/ha. In the second phase (21 d, early-to late-June), the same sheep grazed four 1.5 ha paddocks sequentially at a very light stock density of 7 AU/ha. Dietary composition was assessed through focal bite count observations at the plant functional group level for phase one only, and dietary composition was estimated through fecal DNA metabarcoding (f.DNA) at the plant species level for both phases. Results indicated a uniformly low preference for larkspur (< 1% in diets). There were no significant effects of breed or age on focal bite count observations of plant functional groups (grasses, forbs, and larkspur), nor were there significant effects of breed or age on f.DNA diet proportions of plant functional groups (*p* > 0.05). Stock density did influence focal bite count observations, with higher forb intake (*p* = 0.0004) and lower grass intake (*p* = 0.009) observed at the moderate density compared to the high density. In phase two, grass and larkspur intake decreased while forb intake increased according to f.DNA (*p* < 0.01). These findings suggest that moderate stock density, combined with an understanding of plant phenology, precipitation variability, and animal forage preferences can optimize vegetation and animal performance in adaptive targeted grazing management within this ecosystem.

## 1 Introduction

Targeted grazing, conceptualized as the strategic deployment of specific livestock types at designated times, durations, and intensities to achieve desired vegetation or landscape outcomes (1), is an increasingly important tool for grazing land managers. Targeted grazing takes advantage of complex plant-herbivore interactions that influence plant communities to achieve desired results such as control of individual plant species and removal of fine fuels to mitigate fire risk. It is possible to achieve such goals because livestock can be managed to selectively defoliate the available herbage (2), including undesirable plant species. Targeted grazing provides the integrated advantage of food and fiber production along with control of undesirable plant species (3), distinguishing it from alternative control methods such as herbicides and mechanical removal. However, the efficacy of these strategies is often influenced by environmental conditions, particularly drought, which can alter plant availability and nutritional quality.

Targeted grazing is typically applied to invasive species like leafy spurge (*Euphorbia esula* L.) and spotted knapweed (*Centaurea stoebe* L.), yet its applications extend beyond invasive plant control to encompass various contexts, including the removal of toxic plant species. Larkspurs (*Delphinium* spp.) are a serious toxic plant concern for cattle grazing on rangelands of the western U.S. as they contain numerous diterpenoid alkaloids that block nicotinic acetylcholine (nAch) receptors at the muscular synapse (4, 5). This can result in muscular paralysis, bloat, and death of cattle (6). Sheep exhibit significantly higher resistance to larkspur alkaloids compared to cattle, with a demonstrated tolerance four to six times greater (7, 8).

Sheep at times may preferentially select forbs over grasses due to their distinct prehensile mouth structure and digestive physiology compared to cattle (9, 10). In addition to differences between cattle and sheep on preferential intake of plant functional groups (11), grazing behaviors and dietary preferences differ among sheep breeds. This is particularly evident between hair sheep breeds and fine and medium wool breeds. For example, Dorper sheep exhibit a preference for shrubs (36%) over grasses (64%), in contrast to grass dominated diets of Merinos (86%; 12). Moreover, the U.S. sheep industry exhibits substantial genetic diversity across various breeds, and recent advancements in this domain have enabled producers to enhance productivity and achieve a range of industry objectives (13–15). Given the genetic variability and observed differences in grazing behavior among these breeds, further elucidating these distinctions would be instrumental in optimizing precision-targeted grazing systems.

Although broad trends appear between different species and breeds, grazing is complex and involves a variety of factors beyond inter-animal comparisons. Botanical diversity and composition, past experiences of the animal, recent diet, and post ingestive feedback to plant secondary compounds can influence dietary selection and preference (16–18). These challenges intensify under drought conditions, where climatic extremes further restrict forage availability and quality, requiring more precise management strategies for successful targeted grazing. Precision grazing management integrates precision livestock farming (PLF) techniques to enhance resource efficiency and animal monitoring, with the goal of improving animal performance, nutritional status, welfare, health, and forage utilization (19, 20). In the context of targeted grazing for plains larkspur control, precision grazing management would entail removal of larkspur while ensuring optimal forage availability and protecting both sheep and cattle welfare. Thus, the need to quantify diet selection and preference of grazing animals is important in targeted grazing, especially when co-managing both livestock species with sheep grazing preceding cattle grazing.

A changing climate coupled with land use conversion and changing plant communities increases the need for precision multispecies grazing management for control of noxious plant species (21, 22), and bite counts and fecal DNA barcoding (f.DNA) in tandem are emerging tools that can aid grazing land managers. Integrating multiple grazing species can enhance rangeland conditions by optimizing forage resource utilization, bolstering carrying capacity, and fortifying ecosystem resilience (10, 23). However, managing multispecies grazing under drought conditions presents challenges for targeted grazing strategies. These challenges arise from the need to align plant species and phenological stages with the dietary preferences of both sheep and cattle, a task further complicated by the variable precipitation typical of northern mixed-grass prairie ecosystems. This is particularly evident when the objective of targeted grazing is controlling poisonous plants such as plains larkspur without compromising forage availability for subsequent cattle grazing. Thus, underlying knowledge of forage preferences and dietary selection of sheep targeted grazing is key to designing precision grazing management strategies. Here, our objectives were to investigate the effects of (1) stocking density and (2) breed and age on sheep foraging behavior and dietary preferences for plains larkspur using bite-count and f.DNA methodologies in a northern mixed-grass prairie under drought conditions.

## 2 Materials and methods

### 2.1 Site description

The study was conducted at the United States Department of Agriculture–Agricultural Research Service High Plains Grasslands Research Station located west of Cheyenne, Wyoming, USA (41° 11’ N, 104° 54’ W, elevation 1,930 m a.s.l.). The site is a native, northern mixed-grass prairie comprised of cool-season (C3) grasses and forbs, and warm-season (C4) grasses. The dominant cool-season grasses include western wheatgrass [*Pascopyrum smithii* (Rydb.) Á. Löve], needle-and-thread grass [*Hesperostipa comata* (Trin. & Rupr.) Barkworth], needleleaf sedge [*Carex duriuscula* C.A. Mey.], and prairie junegrass [*Koeleria macrantha* (Ledeb.) Schult]. The dominant warm-season grass is blue grama [*Bouteloua gracilis* (Willd. ex Kunth) Lag. ex Griffiths]. The major forb component is scarlet globemallow [*Sphaeralcea coccinea* (Nutt.) Rydb.], with plains larkspur (*Delphinium geyeri* Greene) the primary toxic plant. Sub-shrubs include spreading buckwheat (*Eriogonum effusum* Nutt.), and prairie sagewort (*Artemisia frigida* Willd.). Primary invasive species are cheatgrass (*Bromus tectorum* L.) and dalmatian toadflax [*Linaria dalmatica* (L.) Mill.].

Average annual precipitation is 385 mm with an average growing season of 128 d (24). Mean annual temperature ranges from an average low of-8°C in December to an average high of 28°C in July (24). Precipitation totals during the study period in 2022 for May and June were 46% (54.1 mm) of the historic average (116.8 mm; 25).

### 2.2 Experimental design

**Figure 1:**
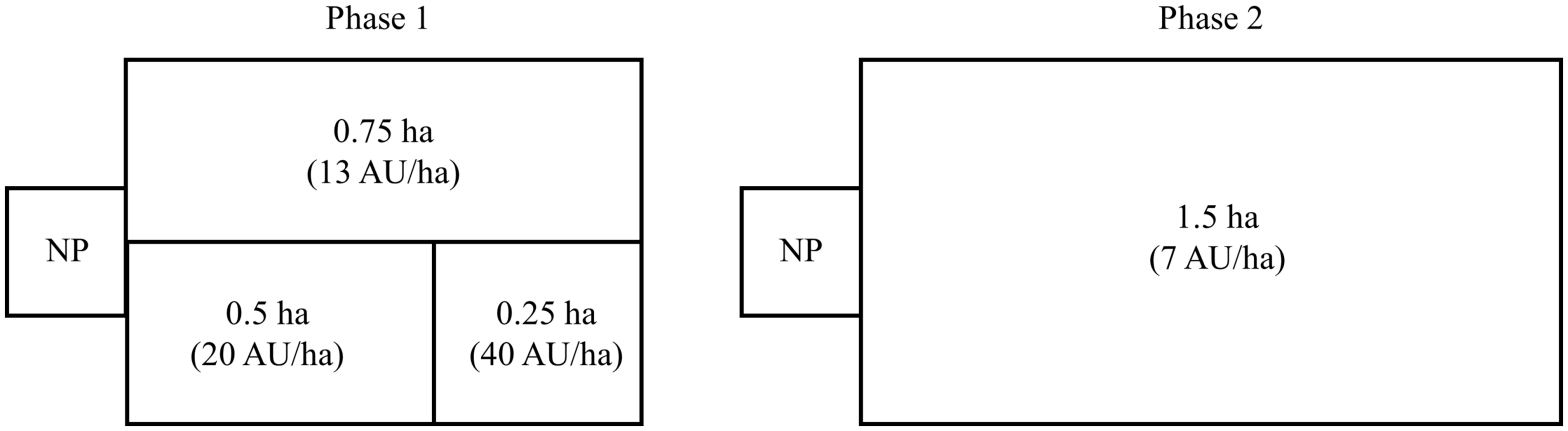
Grazing treatment design for Phase 1 (d 1 – d 20) and Phase 2 (d 20 – d 41). During Phase 1, 157 sheep grazed in three stock density treatments: (1) high, 40 AU/ha (0.25 ha paddock for 1 d), (2) moderate, 20 AU/ha (0.5 ha paddock for 2 days), and (3) light, 13 AU/ha (0.75 ha paddock for 1 d) in four sequential pastures. During Phase 2, the same sheep were grazed in four sequential paddocks at a density of 7 AU/ha (1.5 ha paddocks) for 3–6 days each. For both phases, sheep were held in a common area night pen (NP) from ~1700 h until ~800 h the following morning.

Over a 41-d grazing period (mid-May to late June), 157 sheep, including Rambouillet cull ewes (3-to 6-year-old ewes, *n* = 13, BW 76 kg ± 2.9), Dorper cull ewes (3-to 6-year-old ewes, *n* = 14, BW 47 kg ± 1.8), and Dorper yearling rams (< 1-year-old lambs, *n* = 130, BW 25 kg ± 0.4), were used for two phases of targeted grazing (Figure 1). For the first 20 d (phase 1), electrified woven wire net fencing was used to create three stock density treatments, replicated across four pastures: (1) high, 40 AU/ha (0.25 ha paddock for 1 d), (2) moderate, 20 AU/ha (0.5 ha paddock for 2 d.), and (3) low, 13 AU/ha (0.75 ha paddock for 1 d.). For the second 21 d (phase 2), all sheep were moved to another pasture where they grazed four 1.5 ha paddocks at a very light stock density (7 AU/ha). Fundamentally, the two grazing areas were not different in forage composition and soils; however, differences occurred between the two areas due to phenological stage, biomass, and drought progression. The first two paddocks were grazed for 6 d, but due to forage limitations associated with the drought, grazing duration was reduced to 3 d in the other two paddocks. We recognize that targeted grazing operators typically move livestock when utilization targets are met rather than a set amount of time; however, our focus was on the influence of stocking density on animal diet selection and will be focused on for the present study. At the conclusion of each phase, sheep were kept in temporary paddocks directly adjacent to the treatment paddocks with identical forage composition for two days before weights and fecal samples were collected. Sheep were enclosed each night in a ≈ 127 m^2^ pen (at ~1700 h) and released to graze each morning (at ~0800 h). Sheep were observed daily and had *ad libitum* access to water.

### 2.3 Nutritional composition of grazing diets

Four prominent forage species, representative of nutritional composition of grazing diets, were collected weekly beginning on d 1 and continuing for 8 weeks, including a C3 grass (western wheatgrass), C4 grass (blue grama), and two forbs (scarlet globemallow and plains larkspur). Approximately 30 to 50 g of each forage species were collected adjacent to the grazing treatments and dried in a forced air oven at 60° C for 48 h. The samples were sent to a commercial lab for nutritional and mineral analysis where dry matter (DM), neutral detergent fiber (NDF) and acid detergent fiber (ADF), crude protein, and total digestible nutrients (TDN) were calculated for each sample. NDF and ADF were calculated using ANKOM 2000 and 200/220 fiber analyzers (26, 27; ANKOM Technology, Fairport, NY). Nitrogen content was calculated using a combustion method (28; LECO Corp., St. Joseph, MI), and CP was calculated as 6.25 × N. Trace minerals for each forage sample were calculated using a modified inductively coupled plasma optical emission spectrometry (ICP-OES) method (29; Thermo Fisher Sci. Inc., Waltham, MA). Forage samples were then grouped into early-season and late-season categories comprising weeks 1–4 and 5–8.

### 2.4 Focal bite counts

Focal bite count observations were carried out during phase 1, with two sessions daily (morning and afternoon; 30, 31). Three randomly selected individuals from each of the breed/age groups (3 of each of the Rambouillet ewes, Dorper Ewes, and Dorper lambs) were selected for observation, for a total of 9 observations during each session. For each individual animal, the total number of bites taken was recorded over a span of five minutes, and each bite was categorized into one of three predefined plant functional groups: (1) larkspur, (2) other forbs, and (3) grass or grass-like species. A two-minute interval occurred between observations to allow the observer to select a new individual for focal bite counts. Observations began at around 0800 h and 1600 h in the morning and afternoon, respectively, and continued for approximately 1 h.

### 2.5 Animal performance

Body weights were obtained using a calibrated, electronic scale before (d 0) and after grazing in phase 1 (d 20), and at the end of phase 2 (d 41). Average daily gain (ADG) was calculated for each animal in both grazing phases (20 and 21 d, respectively) and for the study duration (41 d) by taking the total weight (kg) gained divided by the number of days within each respective phase.

### 2.6 Fecal sample collection and DNA barcoding analysis

At each weigh date, 100 sheep were rectal fecal sampled, including all Dorper and Rambouillet ewes (*n* = 27) and 73 randomly chosen Dorper lambs, collecting approximately 30–50 g for each sample. Individual fecal samples were placed in labeled plastic bags and kept in a cooler until they were frozen at-20° C. Fecal samples were randomly chosen for 7 Rambouillet ewes, 10 Dorper ewes, and 23 Dorper lambs at each weight date for f.DNA barcoding analysis. Samples were analyzed at Jonah Ventures Laboratory (Boulder, Colorado, United States), using c-h primers of the trnL intron of plant chloroplast DNA (32). Samples were sequenced and assigned an Exact Sequence Variant (ESV) identification number which is mapped against GenBank reference data. The DNA fragments found in the samples were compared to the reference library to determine the family, and where resolution was sufficient, to genus and/or species. The data was filtered, and we removed any reads with <97% match. We analyzed each family, attempting to narrow down the readings to a single species based on occurrence in the study area. Samples that were labeled to the family, genus, and species level were organized, totaled, and relativized to determine the proportion of each respective plant classification to the total plant material found in the fecal sample. Plant richness and diversity (using *Shannon’s H* Index) were determined for each phase for sheep breeds/ages (33).

### 2.7 Statistical analysis

Bite counts and f.DNA diet proportions were analyzed using mixed linear models in JMP Pro v. 17.0 (SAS Institute, Inc., Cary, North Carolina, United States). Bite proportions and f.DNA diet proportions were compared between breeds (Rambouillet and Dorper ewes) and ages (Dorper ewes and lambs). Bite count percentages of grasses, forbs, and larkspur were converted to proportions and ARCSIN transformed as response variables. Sheep breed/age, stock density, and their interaction were analyzed as fixed effects, while pasture was analyzed as a random effect for phase 1. Fecal DNA percentages of grasses, forbs, larkspur, and other specific species were also converted to proportions and ARCSIN transformed as response variables. Sheep breed/age, grazing phase, and their interaction were analyzed as fixed effects, while individual animal ID was analyzed as a random effect. For all analyses, a 5% level of significance was adopted with *p*-values between 0.5 and 0.1 considered as a tendency.

## 3 Results

### 3.1 Forage nutritional components

The basal diet of sheep grazing under drought conditions in the current study, comprising four representative species—western wheatgrass, blue grama, scarlet globemallow, and plains larkspur—is detailed in Table 1. All forage species demonstrated an increase in NDF percentages, except for blue grama, which saw a decrease in NDF from early-to late-season growth. Similarly, all species showed an increase in ADF percentages, but blue grama remained similar between early and late-season growth. Forbs tended to have lower amounts of NDF and ADF compared to grasses. As fiber components increased later in the season, forage quality decreased, demonstrating lower TDN and CP percentages. Crude Protein and TDN did not change between early-and late-season for blue grama, similar to NDF/ADF values. Forbs generally had greater CP and TDN compared to the grasses, indicating their high nutritional value in grazing diets.

**Table 1:** Nutritional composition of western wheatgrass, blue grama, scarlet globemallow, and plains larkspur collected weekly from mid-May through early-July at the USDA-agricultural research service high plains grasslands research station near Cheyenne, Wyoming, USA.^1^^,^^2^

	Western wheatgrass		Blue grama		Scarlet globemallow		Plains larkspur	
Item	Early	Late	Early	Late	Early	Late	Early	Late
Nutrient composition								
Dry matter, %	93.1 ± 0.1	93.5 ± 0.1	92.4 ± 0.2	92.9 ± 0.1	91.5 ± 0.2	91.6 ± 0.3	92.3 ± 0.2	92.0 ± 0.2
CP, %	17.2 ± 1.3	11.9 ± 0.2	12.5 ± 0.4	12.5 ± 0.7	19.9 ± 0.9	16.5 ± 0.5	18.2 ± 2.8	8 ± 0.7
NDF, %	57.9 ± 2.3	65.6 ± 0.8	68.1 ± 1.1	65.03 ± 1.8	55.8 ± 1.8	76.2 ± 1	25.9 ± 1.1	43.5 ± 3.5
ADF, %	27.1 ± 0.9	33.7 ± 1.1	34.7 ± 0.5	35.7 ± 1.1	22.5 ± 0.2	28.2 ± 1.9	22 ± 0.8	32.2 ± 1.9
TDN, %	71.7 ± 1.1	64.2 ± 1.3	63.1 ± 0.5	61.9 ± 1.3	76.9 ± 0.3	70.4 ± 2.2	77.5 ± 1	65.8 ± 2.1
Mineral composition								
Ca, %	0.4 ± 0.02	0.4 ± 0.01	0.5 ± 0.01	0.5 ± 0.01	1.7 ± 0.1	1.8 ± 0.1	2.7 ± 0.2	2.6 ± 0.3
P, %	0.2 ± 0.01	0.2 ± 0.01	0.2 ± 0.01	0.2 ± 0.01	0.3 ± 0.02	0.2 ± 0.01	0.3 ± 0.03	0.2 ± 0.01
K, %	2.4 ± 0.1	2 ± 0.04	0.7 ± 0.02	0.8 ± 0.1	2.9 ± 0.1	2.6 ± 0.2	3.9 ± 0.1	2.5 ± 0.1
S, %	0.2 ± 0.01	0.2 ± 0.004	0.2 ± 0.01	0.2 ± 0.01	0.4 ± 0.01	0.4 ± 0.01	0.4 ± 0.03	0.2 ± 0.02
Mg, %	0.1 ± 0.006	0.1 ± 0	0.1 ± 0	0.1 ± 0.003	0.3 ± 0.01	0.3 ± 0.01	0.4 ± 0.03	0.2 ± 0.02
Na, %	0.04 ± 0.003	0.04 ± 0.003	0.04 ± 0	0.04 ± 0.003	0.1 ± 0.003	0.04 ± 0	0.04 ± 0.003	0.04 ± 0.003
Fe, mg/kg	123.3 ± 15.8	176.8 ± 10.3	567.3 ± 38.9	627.5 ± 29.1	243.8 ± 34.7	335 ± 87.5	269.8 ± 47.5	185.5 ± 32.9
Mn, mg/kg	32.5 ± 2.6	28.3 ± 1	41 ± 3.4	41.8 ± 3.3	42.3 ± 2.8	61.8 ± 2.7	60.5 ± 1.2	37.3 ± 6
Cu, mg/kg	5.4 ± 0.051	3.6 ± 0.2	5.5 ± 0.5	4.3 ± 0.2	8.7 ± 1	6.9 ± 0.3	7.3 ± 0.7	5 ± 0.5
Zn, mg/kg	23.7 ± 3.0	18.5 ± 0.7	23.5 ± 3.5	18.7 ± 1.8	26.7 ± 4	20.7 ± 1	24.7 ± 1.8	17.1 ± 0.7
Mo, mg/kg	0.8 ± 0.1	0.6 ± 0.1	0.89 ± 0.1	0.9 ± 0.1	0.6 ± 0.03	0.5 ± 0.02	0.9 ± 0.01	0.7 ± 0.1

1 Samples were collected over a period of eight weeks from mid-May through early-July. Early = Week 1–4. Late = Week 5–8.

2 Western wheatgrass (*Pascopyrum smithi*i); blue grama (*Bouteloua gracilis*); scarlet globemallow (*Sphaeralcea coccinea*); plains larkspur (*Delphinium geyeri*).

Mineral composition remained similar between early-and late-season growth, except for Fe, Mn, and Zn which showed variable changes. For example, Mn did not show much of a change between early-and late-season for grasses but forbs showed a larger change in comparison. Scarlet globemallow increased from 42.3 mg/kg ± 2.8 to 61.8 mg/kg ± 2.7 and plains larkspur decreased from 60.5 mg/kg ± 1.2 to 37.3 mg/kg ± 6. Fe content increased for western wheatgrass, blue grama, and scarlet globemallow but decreased for plains larkspur. Zn also demonstrated decreases over the season, with forbs showing a greater decline in comparison to grasses. In summary, the nutrient-level fluctuations observed throughout the grazing study are intended to provide essential context for identifying potential factors influencing dietary selection and subsequent animal performance.

### 3.2 Animal performance

Average daily gain for both phases of this study under drought conditions remained consistent as drought progressed across both phases (0.1 to 0.2 kg/hd/d; Table 2). In phase 1, Dorper ewes had an ADG of 0.2 kg/hd/d, while Rambouillet ewes and Dorper lambs demonstrated an ADG of 0.1 kg/hd/d. For Dorper ewes and Rambouillet ewes, 93 and 92% of the individuals had positive weight gains, respectively, and Dorper lambs only had 66% of the individuals with positive gains. For phase 2, all breed and age classes demonstrated an ADG of 0.1 kg/hd/d. Fewer of the Dorper ewes had positive weight gains (78%) than in phase 1, while 88% of the Dorper lambs and 91% of the Rambouillet ewes had positive gains. Average daily gain across the whole study was 0.1 kg/hd/d for all breed and age classifications, with 86% of the Dorper lambs, 93% of the Dorper ewes, and all the Rambouillet ewes showing positive gains.

**Table 2:** Initial body weight, end body weight, and average daily gain by grazing phase and by 41-d study duration for Dorper ewes, Rambouillet ewes, and Dorper lambs.^1^

	Phase 1			Phase 2			Overall		
BW	D. ewe	R. ewe	D. lamb	D. ewe	R. ewe	D. lamb	D. ewe	R. ewe	D. lamb
Initial, kg	42.6 ± 1.8	74.6 ± 1.9	22.6 ± 0.3	46.2 ± 1.8	76.6 ± 2.1	23.5 ± 0.4	42.6 ± 1.8	74.6 ± 1.9	22.6 ± 0.3
End, kg	46.2 ± 1.8	76.6 ± 2.1	23.5 ± 0.4	47.3 ± 1.9	78.8 ± 2.2	24.9 ± 0.4	47.3 ± 1.9	78.8 ± 2.2	24.9 ± 0.4
ADG, kg/hd/d	0.2 ± 0.03	0.1 ± 0.02	0.1 ± 0.01	0.1 ± 0.02	0.1 ± 0.02	0.1 ± 0.01	0.1 ± 0.02	0.1 ± 0.01	0.1 ± 0.01

1 Phase 1 encompassed a 20-d period, and phase 2 encompassed a 21-d period.

### 3.3 Stock density and focal bite counts

Breed × stock density interactions (*p* > 0.05) did not influence proportions of grass, forb, or larkspur bite counts. Bite counts also did not differ (*p* > 0.05; Table 3) between breeds. The proportions of observed grass and grass-like bites were similar between Dorpers and Rambouillets (97.0% ± 0.8 vs. 94.9% ± 1.3; *p* = 0.24), and proportions of forbs were also similar (2.7%. ± 0.7 vs. 4.3% ± 1.2 forbs; *p* = 0.45). Larkspur bite count proportions were < 1% with values of 0.3% ± 0.1 for Dorper ewes and 0.8% ± 0.3 for Rambouillet ewes (*p* = 0.19).

**Table 3:** Breed and age comparison of bite counts for three plant functional groups: (1) grasses and grass-likes, (2) forbs, and (3) plains larkspur during the mid-May to late June 2022 drought period.^1^^,^^2^

Item	Dorper ewes	Rambouillet ewes	Dorper lambs	Breed *P*-value	Age *P-*value
Graminoids, %	97.0 ± 0.8	94.9 ± 1.3	98.4 ± 0.5	0.24	0.13
Forbs, %	2.7 ± 0.8	4.3 ± 1.2	1.5 ± 0.5	0.46	0.14
Larkspur, %	0.3 ± 0.1	0.7 ± 0.3	0.1 ± 0.1	0.19	0.23

1 Breed comparisons were conducted between the Dorper and Rambouillet ewes, and age comparisons were conducted between the Dorper ewes and lambs.

2 *p-values were determined from the ARCSIN transformed data; arithmetic means are presented as % ± SE.*

Age × stock density interactions (*p* > 0.05) did not influence grass, forb, or larkspur bite counts, nor did age affect these proportions (*p* > 0.05; Table 3). Bite count proportions of grasses were similar between Dorper ewes and lambs (97.0% ± 0.8 vs. 98.4% ± 0.5; *p* = 0.13) as well as bite count proportions of forbs (2.7% ± 0.8 vs. 1.5% ± 0.5; *p* = 0.14). Larkspur selection was also no different between Dorper ewes and lambs (0.3% ± 0.1 vs. 0.1% ± 0.1; *p* = 0.22).

Stock density did influence bite counts for grasses and forbs (*p* < 0.05), with a tendency for less larkspur bites between the first and second day in the moderate density treatment (*p* = 0.07; Table 4). Grass bite count proportions were greater for the high stock density compared to moderate (98.6% ± 0.4 vs. 94.3% ± 1.8; *p* = 0.009) whereas proportions of forb bite counts were greater in the moderate compared to the high stocking density (5.3% ± 1.3 vs. 1.0% ± 0.2; *p* = 0.004).

**Table 4:** Effect of stock density (AU/ha) on observed sheep bite count proportions of plant functional groups.^1,2^

	Stock density				
Item	High	Moderate (day 1)	Moderate (day 2)	Light	*P*-value
Graminoids, %	98.6 ± 0.4^a^	94.3 ± 1.8^b^	97.5 ± 0.8^a^	96.9 ± 0.7^ab^	0.009
Forbs, %	1 ± 0.2^a^	5.3 ± 1.7^b^	2.5 ± 0.8^a^	2.6 ± 0.6^a^	0.004
Larkspur, %	0.5 ± 0.3^a^	0.5 ± 0.2^a^	0.02 ± 0.01^a^	0.5 ± 0.3^a^	0.07

^1^*p*-values were determined from the ARCSIN transformed data; arithmetic means are presented as % ± SE. ^2^Stock Density: High = 0.25 ha (40 AU/ha); Moderate = 0.5 ha (20 AU/ha); Light = 0.75 ha (13 AU/ha). ^a-b^Values within a row containing different superscripts are statistically different at significance level α = 0.05.

### 3.4 Fecal DNA estimated dietary composition

At the plant functional group level, neither breed × phase nor age × phase interactions were observed (*p* > 0.05). Rambouillet and Dorper ewes did not have different dietary compositions for grasses or forbs (*p* > 0.8; Table 5). There was a tendency for age to influence diet composition, though, as Dorper ewe diets had a lower proportion of grasses (38.3% ± 4.2 vs. 46.5% ± 3.1, *p* = 0.09) than lambs, and a greater proportion of forbs (61.5% ± 4.2 vs. 53.4% ± 3.1; Table 5). The proportion of larkspur in sheep diets using f.DNA was minor (< 0.5%) and did not differ between breed (*p* = 0.61) and age classes (*p* = 0.13).

**Table 5:** Breed and age comparison of fecal DNA diet proportions of three plant functional groups: (1) grasses and grass-likes, (2) forbs, and (3) plains larkspur during the mid-May to late June 2022 drought period.^1^^,^^2^

Item	Dorper ewes	Rambouillet ewes	Dorper lambs	Breed *P*-value	Age *P-*value
Graminoids, %	38.3 ± 4.2	36 ± 3.8	46.5 ± 3.1	0.83	0.15
Forbs, %	61.4 ± 4.2	63.9 ± 3.8	53.4 ± 3.1	0.82	0.15
Larkspur, %	0.3 ± 0.1	0.1 ± 0.1	0.1 ± 0.1	0.6	0.14

1 Breed comparisons were conducted between the Dorper and Rambouillet ewes, and age comparisons were conducted between the Dorper ewes and lambs.

2 *p-values were determined from the ARCSIN transformed data; arithmetic means are presented as % ± SE.*

A phase effect was observed for grass, forb, and larkspur proportions (Table 6). The proportion of grasses in diets was 1.4-fold greater (50.1% ± 2.9 vs. 35.1% ± 2.9; *p* = 0.002) in phase 1 compared to phase 2. Conversely, the proportion of forbs in diets was 1.3-fold greater (64.9% ± 2.9 vs. 49.6% ± 2.9; *p* = 0.002). The proportion of larkspur detected in fecal samples was greater in phase 1 (0.2% ± 0.1) than phase 2 (0.004% ± 0.004; *p* = 0.006).

**Table 6:** Sheep fecalDNA (f.DNA) metabarcoding diet proportions of plant functional groups compared between grazing phases.^1^

	Phase		
Item	Phase 1	Phase 2	*P*-value^2^
Graminoids, %	50.1 ± 2.9	35.1 ± 2.9	0.002
Forbs, %	49.6 ± 2.9	64.9 ± 2.9	0.002
Larkspur, %	0.2 ± 0.1	0.004 ± 0.004	0.006

1 *p-values were determined from the ARCSIN transformed data; arithmetic means are presented as % ± SE.*

2 *p-values < 0.05 are significant.*

Age × phase interactions influenced proportions of western wheatgrass and scarlet globemallow in sheep diets. The proportion of western wheatgrass detected in Dorper lamb diets was 1.9-fold greater in phase 2 than phase 1 (17.2% ± 2.1 vs. 9.1% ± 2.0; *p* = 0.001), but this difference did not occur for Dorper ewes (*p* > 0.9). Likewise, the proportion of scarlet globemallow detected in Dorper lambs was also greater in phase 2 (4.4% ± 1.4) compared to phase 1 (0.4% ± 0.2; *p* = 0.0006), with no difference for Dorper ewes (*p* > 0.97).

No differences were observed between breeds at the species level using f.DNA (*p* > 0.15; Table 7), whereas proportions differed between Dorper ewes and lambs for several plant species (Table 8). Proportions trended higher for Dorper lambs than ewes for blue grama (2.8% ± 0.7 vs. 0.9% ± 0.3), mountain bladderpod (1.2% ± 0.4 vs. 0.3% ± 0.1), and cheatgrass (8.9% ± 1.7 vs. 5.3% ± 1.5; 0.05 < *p* < 0.1), while needleleaf sedge exhibited a significant response (0.4% ± 0.1 vs. 1.0% ± 0.2; *p* = 0.01).

**Table 7:** Diet comparison of specific plant species in sheep diets between breeds across phases using fecal DNA (f.DNA) metabarcoding.^1^

	Sheep breed		
Species, % of total	Rambouillet ewe	Dorper ewe	*P*-value^2^
C3 graminoids			
*Hesperostipa comata* (needle and thread)	18.4 ± 4.7	14.8 ± 2	0.37
*Carex duriuscula* (needleleaf sedge)	0.6 ± 0.2	0.4 ± 0.1	0.25
*Pascopyrum smithii* (western wheatgrass)	8.7 ± 1.5	14.3 ± 2.8	0.17
C4 graminoids			
*Bouteloua gracilis* (blue grama)	0.9 ± 0.5	0.9 ± 0.3	0.79
Forbs			
*Sphaeralcea coccinea* (scarlet globemallow)	0.5 ± 0.2	1.2 ± 0.4	0.25
*Lesquerella montana* (mountain bladderpod)	1.0 ± 0.7	0.30 ± 0.1	0.40
Problem rangeland plants			
*Bromus tectorum* (cheatgrass)	4.0 ± 1.5	5.3 ± 1.5	0.25
*Euphorbia* spp. (spurge spp.)	0.2 ± 0.1	2.3 ± 1.4	0.25
*Linaria dalmatica* (dalmatian toadflax)	Not detected	0.4 ± 0.3	0.17

1 *p-values were determined from the ARCSIN transformed data; arithmetic means are presented as % ± SE.*

2 *p-values < 0.05 are significant.*

**Table 8:** Diet comparison of specific plant species in sheep diets between Dorper ewes and lambs across phases using fecal DNA (f.DNA) metabarcoding.^1^

	Sheep age		
Species, % of total	Dorper ewe	Dorper lamb	*P*-value^2^
C3 graminoids			
*Hesperostipa comata* (needle and thread)	14.8 ± 2	16.6 ± 1.4	0.41
*Carex duriuscula* (needleleaf sedge)	0.4 ± 0.1	1.0 ± 0.2	0.01
*Pascopyrum smithii* (western wheatgrass)	14.3 ± 2.8	13.2 ± 1.5	0.65
C4 graminoids			
*Bouteloua gracilis* (blue grama)	0.9 ± 0.3	2.8 ± 0.7	0.07
Forbs			
*Sphaeralcea coccinea* (scarlet globemallow)	1.2 ± 0.4	2.4 ± 0.8	0.66
*Lesquerella montana* (mountain bladderpod)	0.3 ± 0.1	1.2 ± 0.4	0.06
Problem rangeland plants			
*Bromus tectorum* (cheatgrass)	5.3 ± 1.5	8.9 ± 1.7	0.09
*Euphorbia* spp. (spurge spp.)	2.3 ± 1.4	0.7 ± 0.4	0.18
*Linaria dalmatica* (dalmatian toadflax)	0.4 ± 0.3	0.5 ± 0.2	0.46

1 *P-values determined from the ARCSIN transformed data; arithmetic means are presented as % ± SE.*

2 *P-values < 0.05 are significant.*

### 3.5 Fecal DNA plant richness and diversity

Plant richness, encompassing the observed number of plant species in diets, was not influenced by breed × phase nor age × phase interactions, nor by breed or age individually (*p* > 0.4). Phase did influence plant richness with higher richness in phase 2 (11 species) compared to phase 1 (7 species; *p* < 0.04; Table 9).

**Table 9:** Species richness and Shannon’s diversity comparisons between Rambouillet ewes, Dorper ewes, and Dorper lambs, and between grazing phases 1 and 2 determined through fecal DNA (f.DNA) metabarcoding.

	Breed		*P*-value^2^		
Item:	Dorper ewe	Rambouillet ewe	B	P	B × P
Species richness			0.85	0.04	0.69
Phase 1	7.5 ± 1.1^a^	7.9 ± 1.3^a^			
Phase 2	10.8 ± 1.1^b^	10.0 ± 1.3^b^			
Shannon’s diversity^1^			0.42	0.08	0.8
Phase 1	1.3 ± 0.1^a^	1.2 ± 0.1^a^			
Phase 2	1.5 ± 0.1^a^	1.4 ± 0.1^a^			
	Age		*P* – value^3^		
Item:	Dorper ewe	Dorper lamb	A	P	A × P
Species richness			0.73	< 0.0001	0.44
Phase 1	7.5 ± 1.1^a^	7.2 ± 0.7^a^			
Phase 2	10.8 ± 1.1^b^	12.0 ± 0.7^b^			
Shannon’s diversity^1^			0.03	< 0.0001	0.04
Phase 1	1.3 ± 0.1^a^	1.3 ± 0.1^a^			
Phase 2	1.5 ± 0.1^a^	1.8 ± 0.1^b^			

1 Shannon’s index is calculated as $H^{\prime }=-\sum_{i=1}^{s}p_{i}\ln p_{i}$ where pi is the proportion of the ith species, s is species richness, and H′ is diversity.

2 B = breed; P = grazing phase.

3 A = Age; P = grazing phase.

^a-b^ Values within a row containing different superscripts are statistically different at significance level α = 0.05.

Age × phase interaction did affect plant diversity in sheep diets (*p* = 0.03). Dorper lambs exhibited greater plant diversity in diets during phase 2 compared to phase 1 (1.8 ± 0.1 vs. 1.3 ± 0.1; *p* < 0.0001), but Dorper ewes did not (*p* > 0.35). In addition, Dorper lambs exhibited higher plant diversity compared to Dorper ewes in phase 2 (1.8 ± 0.1 vs. 1.5 ± 0.1; *p* < 0.0001). Breed did not affect plant diversity in diets for either phase (*p* > 0.2).

## 4 Discussion

Under drought conditions neither sheep breed nor age influenced bite count proportions or f.DNA diet proportions of grasses, forbs, and plains larkspur. The lack of breed and age differences for selection of plains larkspur concurs with low palatability of this species during early growth stages (34–36). The timing of targeted grazing in the current study (late May to June) coincided with the early vegetative phenological stage of plains larkspur. Moreover, desirable cool-season (C3) grasses expressed their growth during this period which further reduced desirability of plains larkspur. Pfister et al. (37) recommended that sheep could be herded onto discreet patches of larkspur to reduce larkspur abundance (i.e., targeted grazing), while acknowledging that when larkspur is more uniformly distributed across a pasture, a herding strategy for larkspur reduction is more limited.

Due to their purported generalist grazing tendencies (12), Dorper sheep were initially hypothesized to exhibit less avoidance for plains larkspur and greater forb components in their diets, although their prior experiences and whether they had been exposed to larkspur previously was not known. Drought conditions and lack of biomass across all plant functional groups likely constrained expression of divergent grazing behavior across breeds. Periodic drought has been shown to decrease species richness and above-ground plant biomass in grasslands (38–40), and in the current study where precipitation was 46% of the historic average, biomass was 1,215 kg/ha compared to 1,547 kg/ha under normal conditions (36). Under ideal experimental conditions characterized by high plant biomass and forb plant components, differences in dietary preferences might be more pronounced (12). Thus, it’s likely that under drought conditions where biomass is limited that breed differences matter less than timing, duration, and stock density. Nonetheless, this study offers insights into grazing dietary selection in drought conditions common to the U.S. high plains region, which are becoming increasingly common in a climate change scenario. Future research on sheep breed dietary preference merits further investigation, especially under more conducive grazing conditions of adequate biomass and plant species diversity.

Previous research at this site observed significant reductions in larkspur density (plants/m^2^) at moderate and high stocking densities but not light stocking density with sheep grazing. Stock density was more influential than breed or age of sheep in the current study, which is similar to grazing behavior differences (e.g., straighter lines of grazing; 41) and lower weight gains (42) seen with cattle and high stock density; however, standing crop estimates also indicated that sheep grazing could cause significant reductions in available biomass, especially during a drought year (36). In arid and semi-arid rangelands, adaptive grazing to ensure adequate recovery of range condition through plant regrowth is easier achieved when grazing pressure is reduced (43–45). Therefore, a lighter stocking rate may be more effective in a multispecies grazing scenario. The lack of differences in observed bite counts of grasses and forbs between the high and light stock density was unexpected, as we initially hypothesized that sheep would select for more forbs and fewer grasses at a light animal density. Though this was partially true because we saw differences between the high density and d 1 of the moderate density treatments, we attributed the lack of differences between high and light densities to the stocking rate of our high and moderate density treatments. Both treatments were grazed at the same stocking rate (40 Animal Unit Days (AUD)/ha), even though stocking density differed between the two treatments. Hunger resulting from multiple days of high grazing pressure and inadequate forage availability likely constrained the selectivity of these animals when entering the light density treatment. A crucial aspect of targeted sheep grazing is that it will require high capacity in adaptive decision making to respond to changes in climate and forage availability within and across years, when applied in general as well as for control of larkspur. For example, utilizing predictive grazing tools such as Grass Cast and U.S. Drought Monitor combined with ground-based assessments of animal utilization and range condition can assist managers in making adaptive decisions (46).

Added complexity for those wanting to use sheep targeted grazing for plains larkspur control is the context associated with ensuring leaving sufficient forage for subsequent cattle grazing. This requires the sheep to actively select for larkspur while leaving sufficient grass biomass for cattle. Optimizing both sheep and cattle grazing on the same area of land will require substantial management acumen for land managers, principally knowledge of ecological sites, plant diversity, and forage preferences of grazers. For example, conflicts in outcomes with cattle and sheep grazing the same area depends on which species grazes first as well as the extent of dietary overlap. Cattle grazing followed by sheep in grass-dominated subirrigated pastures was beneficial by increasing available grazing days by >50% because it facilitated grass regrowth during the peak growing season (47). Conversely, in mixed perennial ryegrass (*Lolium perenne*)/white clover (*Trifolium repens*) swards, when sheep grazed first, more reproductive grass stems and lower white clover abundance occurred (48). In the context of poisonous plants for cattle, grazing sheep first is necessary for an optimal sequence in these rangelands which contain larkspur. Grazing is a complex interaction between herbivores and plant communities, depending on more factors than species and breed differences alone. Botanical diversity of the plant community and physiological status of the animal have been shown to influence foraging behavior, which were not addressed in the current study (16, 18). Furthermore, it is not known what plant species this cohort of sheep had dietary exposure to prior to the study, which could be important for targeted grazing (17). Subsequent research could further delineate the selectivity of cattle and sheep in this system based on plant diversity, physiological status, and recent environments of the animals being employed which were not accounted for in the study design.

Knowledge of the species consumed by grazing animals with targeted grazing is fundamental in determining the efficacy of this grazing management strategy (49, 50) and has important implications to precision livestock management in extensive systems. Bite counts are easy to conduct and require minimal equipment but are often conducted on a small subset of the grazing animals (50, 51). The bite count methodology tends to overestimate grasses and underestimate forbs (31). In contrast, f.DNA methods can be used on a much larger set of grazing animals through fecal sampling; however, local knowledge of the plant species for comparative analyses of the laboratory-derived information is needed. The methodology has limitations with accuracy of data from fed-diet studies, including misdetection of species and underrepresentation of warm-season (C4) plants in the diets compared to known fed proportions (52). Despite the limitations, the methodology has been successfully used in the detection of poisonous plants (53, this study).

Neither bite-count nor f.DNA proportions revealed discernable differences between sheep breed or age at the plant functional group level, but the ability to observe specific plant species as well as plant richness and diversity using f.DNA revealed additional insights. Dorper lambs demonstrated significantly higher proportions of short-statured, fine-leaved grasses (blue grama and cheatgrass) compared to Dorper ewes. Due to their small mouth and body size, the lambs will select shorter plants over taller ones (54) and plants with a high leaf:stem ratio (55) for higher dietary quality. The greater botanical diversity selected by Dorper lambs compared to ewes in phase 2 indicates that the lambs grazed more species. It is important to recognize that the Dorper sheep came from the same source flock, so it’s possible they would have been exposed to similar grazing lands with similar plant compositions prior to the study, suggesting there was a naivety component. That is, they had not attained the same experiential knowledge of grazing–and were therefore more exploratory (56).

## 5 Conclusion

Diet selection and composition of Rambouillet and Dorper ewes differed little with targeted grazing within a northern mixed-grass prairie during a drought period in late spring/early summer 2022. Minor differences were observed between Dorper ewes and lambs for several plant species and diet diversity according to f.DNA. Drought-induced reductions in plant richness, diversity, and aboveground biomass likely constrained the expression of breed and age selectivity for plains larkspur. High stock density was more influential under these drought conditions, although implementing high-intensity, short-duration grazing of smaller paddocks (e.g., 0.25 to 0.75 ha) may be impractical for managers on a large scale. Herding, both traditional and evolving virtual herding systems (i.e., virtual fencing), might provide alternative ways to increase stock density, but tradeoffs with impacting non-target plant species and available forage for cattle grazing are important to consider. Future research should aim to understand the mechanisms driving animal diet selection and overlap across species, particularly under more favorable grazing conditions with greater precipitation, to improve precision sheep-targeted grazing strategies.

## Data Availability

The raw data supporting the conclusions of this article will be made available by the authors, without undue reservation.
